# Westminster Hospital

**Published:** 1830-02

**Authors:** 


### HOSPITAL REPORTS.

### WESTMINSTER HOSPITAL.

In our Number for December, we gave the dissection report of the limb and tumor of Amelia Becket. On opening the body fourteen days afterwards, the same disease appeared to have affected the lungs and the liver. There were several tubercles on the surface of the lungs, some of the size of a walnut, containing a whitish medullary matter; one of them adherent to the ribs. Those on the liver were smaller, and not so far advanced. This dissection renders it doubtful whether the operation would have been successful, if it had been done at an earlier period. The disease being evidently constitutional, a question may arise as to the probability of its having been so originally, or as to its having become so from the continuation of disease.

The following case is interesting on account of the fact of vomiting having accompanied so well marked a case of compression; a point to which Mr. Guthrie drew the attention of the students in a particular manner, as being one which many persons were induced to dispute, and some to deny.

Thomas Sullivan, aged fifteen, admitted December 4th: he had fallen from a scaffolding forty feet high, and was insensible, his head swollen, the scalp torn, the bones broken and depressed, and the brain coming through the external wound; the breathing stertorous, pulse imperceptible; bleeding from the right ear, and, shortly after his admission, vomiting. Mr. Guthrie, on his arrival, sawed off, with Hey's saw, two points of bone; and then raised the depressed portion, which however immediately returned to nearly its former state, on the removal of the levator, the bone appearing to be bent. This was obviated by keeping the bone steadily elevated for several minutes by the end of the lenticular, when the bend was found to have been overcome; a use for that instrument to which we have never seen it before applied. The fracture was now found to have separated the mastoid process from the body of the temporal bone, and the brain was also coming through the ear. The case was considered hopeless, and the boy died a few hours afterwards.

